# Molecular characterization of Kawasaki disease subgroups using cell-free RNA profiling

**DOI:** 10.1038/s41598-025-15843-7

**Published:** 2025-08-14

**Authors:** Conor J. Loy, Hao Wang, Jihoon Kim, Chisato Shimizu, Joan Lenz, Emma Belcher, Adriana H. Tremoulet, Jane C. Burns, Iwijn De Vlaminck

**Affiliations:** 1 Meinig School of Biomedical Engineering, Cornell University, Ithaca, NY 14850 USA; 2 Department of Pediatrics, Kawasaki Disease Research Center, University of California San Diego, La Jolla, San Diego, CA 92093 USA; 3 Department of Biomedical Informatics and Data Science, Yale School of Medicine, New Haven, CT 06510 USA; 4 Department of Pediatrics, Rady Children’s Hospital-San Diego, San Diego, CA 92123 USA

**Keywords:** Cell-free RNA, Kawasaki disease, Pediatric, Liquid biopsy, Biomarkers, Vasculitis

## Abstract

Kawasaki disease is a pediatric vasculitis and the leading cause of acquired heart disease in children. The heterogeneous clinical presentation of Kawasaki disease complicates diagnosis and treatment, highlighting the need for molecular signatures to stratify patients into subgroups to better understand pathogenesis. We performed plasma cell-free RNA sequencing on samples from 98 patients diagnosed with Kawasaki disease, 86 febrile children (62 viral infection, 24 bacterial infection), and 5 healthy children. The Kawasaki disease patients were previously classified into one of four clinical subgroups using 14 clinical variables. Significant differences were observed in cell-free RNA transcript abundance, pathway enrichment scores, and cell type-of-origin fractions, including differences in hepatocyte injury, mitochondrial dysfunction, platelet activation, and developmental markers. This study demonstrates the utility of cell-free RNA to characterize Kawasaki disease subgroups at a molecular level. Cell-free RNA profiles may be used as biomarkers for Kawasaki disease stratification and offer new insight into the pathogenesis of specific KD phenotypes.

**Supplementary Information:**

The online version contains supplementary material available at 10.1038/s41598-025-15843-7.

## Introduction

Kawasaki Disease (KD) is an acute vasculitis that predominantly affects children under the age of five^1^. Patients can present with a variable constellation of clinical signs, including fever, rash, conjunctival injection, oropharyngeal erythema, extremity edema, and cervical lymphadenopathy^1^. KD is the leading cause of acquired heart disease in children worldwide with the development of coronary artery aneurysms (CAA) in 25% of untreated children^1^. The heterogeneous presentation of clinical signs and outcomes makes KD difficult to study, particularly when identifying biomarkers for diagnosis and developing targeted therapies^2^. Understanding the molecular profiles of KD subgroups offers a promising avenue for individualized therapies and improved patient outcomes.

Recent work by Wang et al. utilized a data-driven approach to identify four subgroups of KD patients based on 14 clinical and laboratory features^3^. Key differing features included hepatic enzyme elevation, age, immune cell counts, rash, cervical lymphadenopathy, coronary artery aneurysm rates, and response to IVIG treatment, suggesting heterogeneity in the underlying molecular pathology. Differential abundance of proteins and distinct seasonality trends were also observed among the four subgroups, raising the question of different etiologies. These subgroups offer promising insights into KD patient heterogeneity, but further characterization and validation of these subgroups is needed.

Plasma cell-free RNA (cfRNA) is released through cell death and active release from both circulating cells and cells residing in tissues, and is thus an ideal analyte for characterizing the heterogeneity observed in KD patients^4^. Previous work has shown that cfRNA has diagnostic potential in the setting of tuberculosis, preeclampsia, cancer, and transplantation, and can be used to track health over time, such as during immunotherapy treatment and space flight^5–11^. Recently, we demonstrated the diagnostic potential of cfRNA to differentiate KD from Multisystem Inflammatory Syndrome in Children (MIS-C) and other febrile conditions in children^5^. However, the potential of cfRNA to characterize disease subtypes has not been previously explored.

In this study, we implemented molecular characterization via plasma cfRNA sequencing for 98 KD patients and 91 pediatric controls (viral infection, bacterial infection, and healthy). We compared previously described subgroups of KD at three levels of cfRNA analysis: transcript abundance, pathway enrichment, and cell type-of-origin fraction. Using these measurements, we detected significant differences between groups and discovered novel molecular features. We also performed unsupervised clustering of the cfRNA samples to explore *de novo* molecular subtyping of KD.

## Results

### Comparison of KD subtypes using CfRNA

**Fig. 1:**
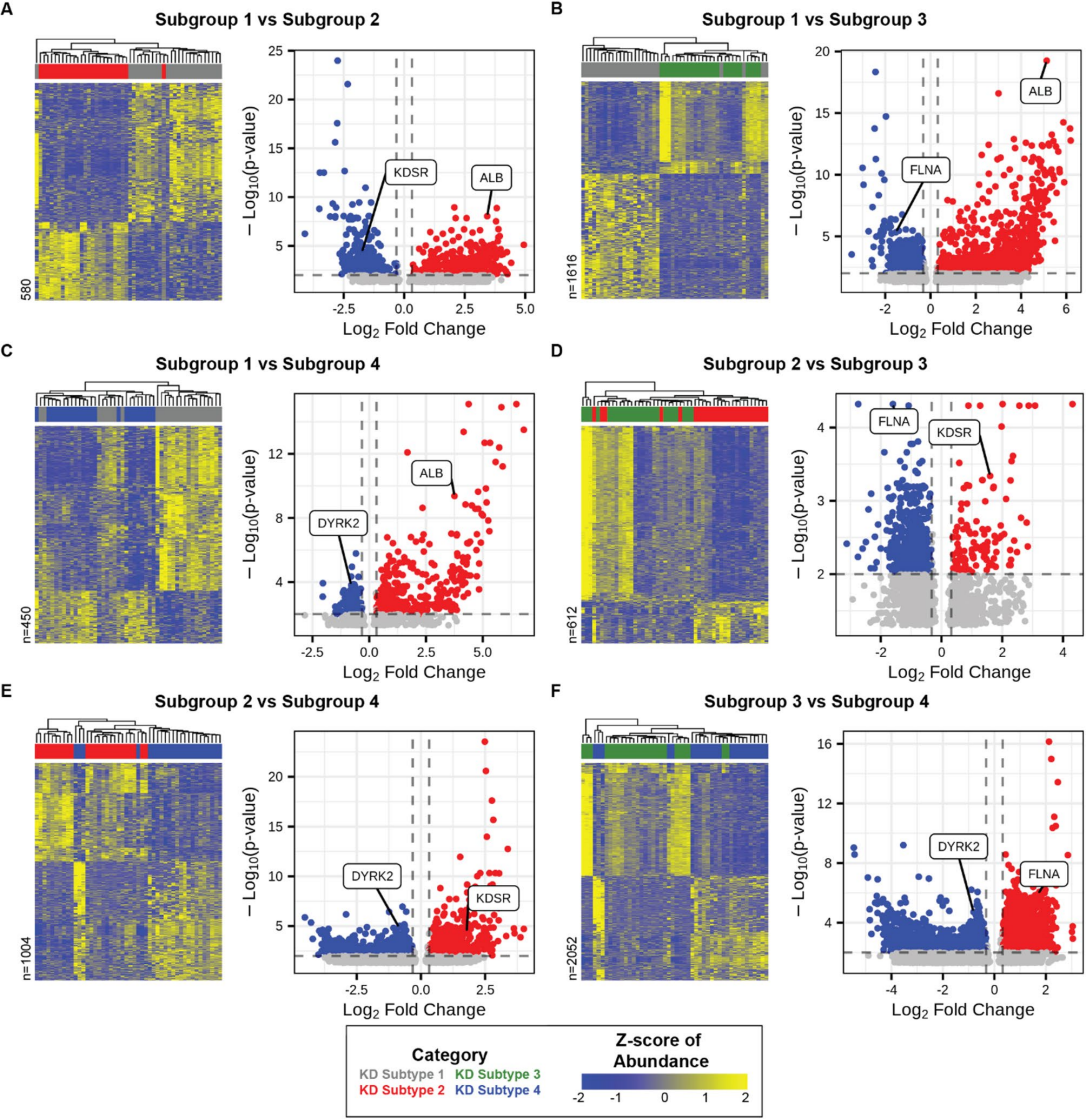
Pairwise RNA transcript comparisons between KD subtypes. Differential abundance analyses between KD subtypes are shown for: (**A**) Subtype 1 vs. 2, (**B**) Subtype 1 vs. 3, (**C**) Subtype 1 vs. 4, (**D**) Subtype 2 vs. 3, (**E**) Subtype 2 vs. 4, and (**F**) Subtype 3 vs. 4. Heatmaps display the top differentially abundant RNA transcripts (DESeq2, Benjamini-Hochberg adjusted p-value < 0.01, absolute fold change > 1.25). Volcano plots depict all RNA transcripts, with transcripts more abundant in the first listed subtype shown in red, and those less abundant in blue.

We first measured differences in cfRNA profiles between KD subgroups by pairwise differential abundance analysis, comparing each pair of subgroups (Methods). Each comparison yielded hundreds to thousands of differentially abundant RNA transcripts (Benjamini-Hochberg adjusted p-value < 0.01, absolute fold change > 1.25) (Fig. 1A-F, Supplementary File 1). We observed many RNA transcripts that were consistently more abundant in one subgroup compared to the others. For each subgroup, the transcripts with the most significantly altered abundance across comparisons were albumin (*ALB*) for subgroup 1, 3-Ketodihydrosphingosine Reductase (*KDSR*) for subgroup 2, filamin A (*FLNA*) for subgroup 3, and Dual Specificity Tyrosine Phosphorylation Regulated Kinase 2 (*DYRK2*) for subgroup 4 (Benjamini-Hochberg adjusted p-value: *ALB* = 3.2e-9, *KDSR* = 1.8e-4, *FLNA* = 1.8e-5, *DYRK2* = 6.4e-5). We also found increased levels of *DYRK2* in febrile control (FC) samples from patients under four years of age, indicating that the increased abundance in KD subgroup 4 is likely due to the younger ages of patients in this subgroup (Figure S1A). We next reanalyzed every pairwise comparison with illness day of sample collection included as a covariate to account for differences in sample collection days among subgroups (Figure S1B-H). The results were largely unchanged, with the only notable exception in the comparison of Subgroup 2 vs. Subgroup 3 that resulted in fewer significant transcripts. This comparison had showed only modest molecular differences between these groups and a significant difference in illness day of sample collection, thus making distinguishing biological signal from sampling timing difficult. These observations highlight the molecular differences between KD subgroups and confirm that they are largely independent of collection timing.

Given the significant differences between subgroups, we next compared each subgroup to samples from patients with adenovirus infection (*n* = 20), given that adenovirus infection shares many clinical features with KD. We observed substantially different numbers of differentially abundant RNA transcripts in each subgroup (subgroup 1 = 789, subgroup 2 = 577, subgroup 3 = 923, subgroup 4 = 319; Benjamini-Hochberg adjusted p-value < 0.01 and absolute fold-change > 1.25) (Figure S2 A-D). We also found 30 RNA transcripts that were differentially abundant in adenovirus compared to all four subgroups, and those elevated in patients with acute adenovirus infection were largely from Type 1 interferon related genes (Table S3, Figure S2 E-F).

### Pathway enrichment analysis

**Fig. 2:**
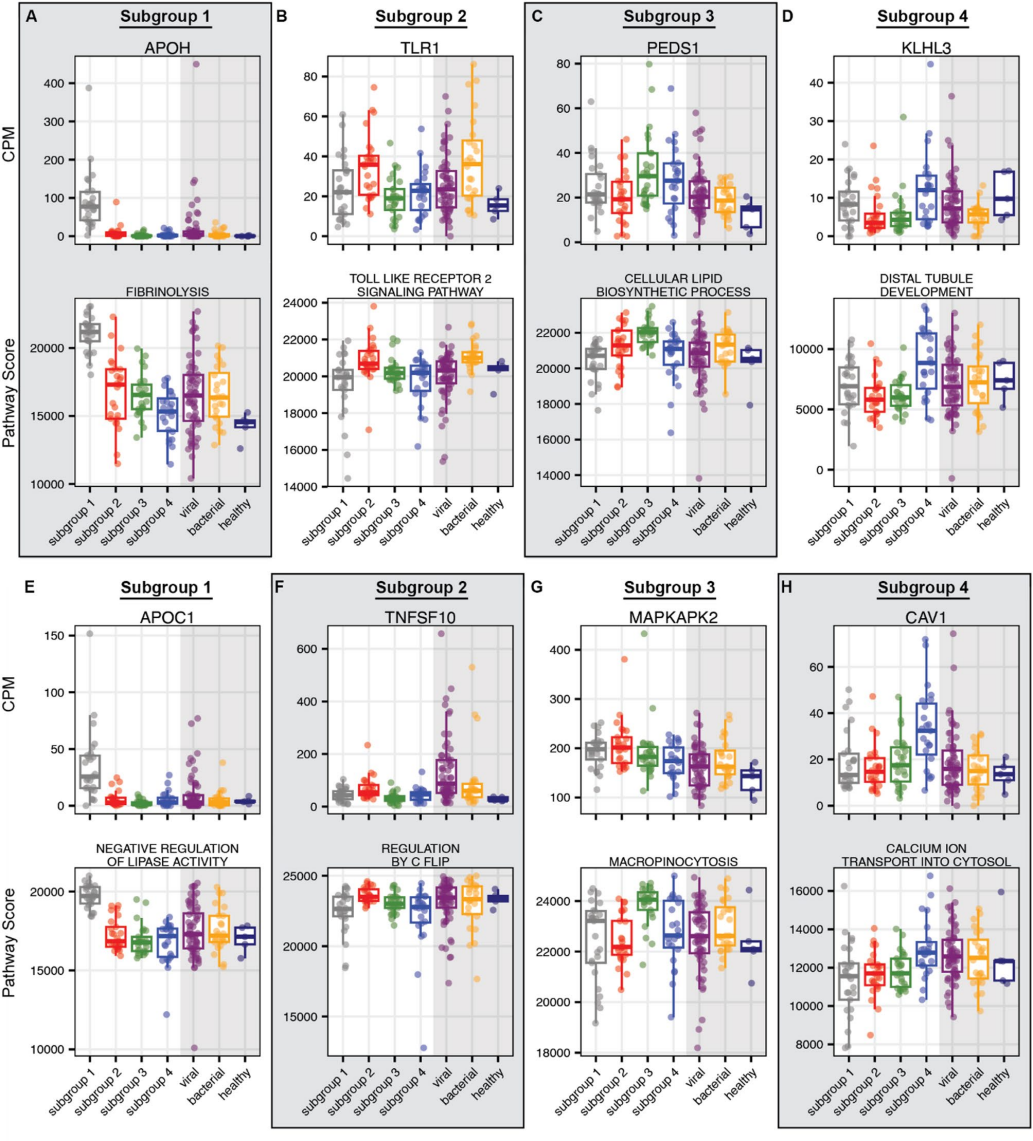
Pathway enrichment analysis. Single-sample Gene Set Enrichment Analysis was performed to compare pathway enrichment scores between each KD subtype and all others. For each subgroup, the top most significantly enriched pathway (**A-D**) is shown along with the RNA transcript most significantly associated with that pathway. Additionally, the second most significantly enriched pathway that does not share members with the first is shown, along with its top RNA transcript (**E-H**). The gray shaded portions of the plots highlight the three control groups. Abbreviations: CPM = Counts Per Million, APOH = Apolipoprotein H; TLR1 = Toll Like Receptor 1; PEDS1 = Plasmanylethanolamine Desaturase 1; KLHL3 = Kelch Like Family Member 3; APOC1 = Apolipoprotein C1; TNFSF10 = TNF Superfamily Member 10; MAPKAPK2 = MAPK Activated Protein Kinase; CAV1 = Caveolin 1.

We next investigated the utility of cfRNA to characterize the KD subgroups using ssGSEA (Methods). We performed one-versus-all comparisons of pathway scores to identify subgroup-specific enriched pathways (Supplementary File 2, Methods). The two most enriched pathways between subgroups that did not share any genes, as ranked by adjusted p-value, were Subgroup 1: Fibrinolysis and Negative Regulation of Lipase Activity; Subgroup 2: Toll Like Receptor 2 Signaling Pathway and Regulation by C Flip; Subgroup 3: Cellular Lipid Biosynthetic Process and Macropinocytosis; Subgroup 4: Distal Tubule Development and Calcium Ion Transport into Cytosol (Fig. 2A-H). The abundance of the most significant gene member of each pathway reflected the pathway enrichment scores (Fig. 2A-H).

Pathways enriched in subgroup 1 were generally liver-related (Fibrinolysis, Negative Regulation of Lipase Activity, Recycling of Bile Acids and Salts) and driven by albumin and Apolipoprotein-family genes (*APOH*,* APOC1*-3, Table S4). We also observed an enrichment of pathways relating to other tissues, including the gastrointestinal tract (Smooth Muscle Cell Apoptotic Process, Intestinal Cholesterol Absorption) and pancreas (Regulation of Gene Expression in Beta Cells, Fig. 2A and E, Supplementary File 2). However, there was overlap of these pathways with Apolipoprotein-family genes, indicating these pathways were potentially also indicative of liver damage.

For subgroup 2, we observed an enrichment of antigen detection pathways (Toll Like Receptor 2 Signaling, Regulation of MHC Class II Biosynthetic Process) driven by genes *TLR1*, *STAT1*, and *JAK2* (Table S4). Interestingly, we also found that subgroup 2 transcriptomes were enriched for cell death related pathways (Regulation by C Flip, Caspase Activation via Death Receptors in the Presence of Ligand) driven by *TNFSF10*, and enrichment of mitochondrial metabolic processes (Glutathione synthesis and recycling, folic acid containing compound metabolic process) driven by genes *ATIC*, *MTHFD2*, and *GGT7* (Table S4). We observed similar trends in the control groups of patients with viral and bacterial infection (Fig. 2B and F, Supplementary File 2).

Subgroup 3 transcriptomes were enriched with genes related to Lipid biosynthesis (Cellular Lipid Biosynthetic Process, Sphingolipid De Novo Biosythesis) driven by *PEDS1* and *PPM1L* (Table S4). We also observed an enrichment of actin-dependent functions (Micropinocytosis, Positive Regulation of Actin Nucleation) driven by *MAPKAPK2*, *WASF1*, and *NCKAP1* (Fig. 2C and G, Supplementary File 2, Table S4).

Lastly, in subgroup 4 we observed significantly enriched pathways related to differentiation and development (Distal Tubule Development, Somatic Stem Cell Division) driven by *KLHL3*, *DLG5*, and *LBH* (Table S4). We also observed an enrichment of ion transport related pathways (Positive Regulation of Calcium Ion Transport Into Cytosol, Renal Sodium Ion Transport) driven by *CAV1* and *KLHL3* (Fig. 2D and H, Supplementary File 2, Table S4). We compared these levels to FC samples from patients under four years of age and found that the observed pathway enrichment in KD subgroup 4 is likely not due simply to the younger age of the patients (Figure S3A-B).

### Cell type-of-origin

**Fig. 3:**
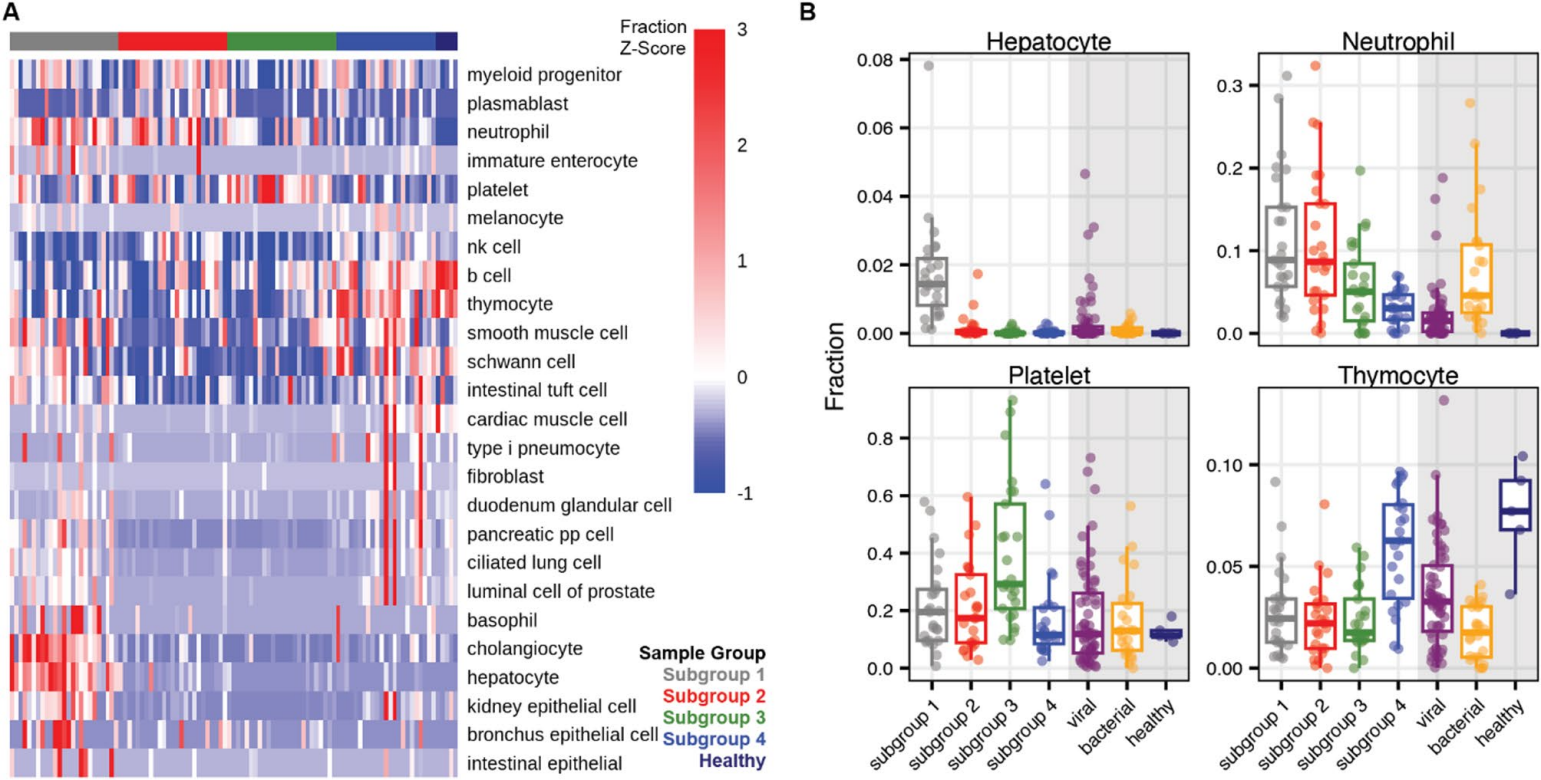
Deconvolution of the cfRNA cell types of origin. (**A**) Estimated cell type-of-origin fractions are shown for cell types with significantly different means across KD subtypes (ANOVA, Benjamini-Hochberg adjusted p-value < 0.05). The heatmap represents the Z-scores of the fractions for each cell type across subgroups and healthy controls. (**B**) Hepatocyte, neutrophil, platelet, and thymocyte derived cfRNA fractions across all sample groups (age distribution in years in each sample group [mean +/- standard deviation]: subgroup 1 = 5 +/- 3.4, subgroup 2 = 4.8+/-2.6, subgroup 3 = 3.8 +/- 1.6, subgroup 4 = 2 +/- 1.5, viral = 5.8 +/- 3, bacterial = 10 +/- 5, healthy = 5 +/- 0.9). Gray shaded portion of plots highlight the three control groups.

We performed cfRNA deconvolution to estimate the cell types-of-origin of cfRNA, allowing us to profile cellular damage and death, which are key aspects of KD pathology. To achieve this, we applied the BayesPrism deconvolution algorithm with the Tabula Sapiens scRNA-seq atlas as a reference (Methods). We then used ANOVA to compare the mean proportion of each cell type across subgroups, identifying significant differences in 39 cell types (Benjamini-Hochberg adjusted p-value < 0.05) (Fig. 3).

In Subgroup 1, we observed an increased proportion of cfRNA derived from hepatocytes and intrahepatic cholangiocytes, along with increased cfRNA from neutrophils, basophils, intestinal stem cells, and kidney epithelial cells (Fig. 3B). Subgroup 2 was characterized by higher cfRNA levels from neutrophils and myeloid progenitor cells (Fig. 3B). Subgroup 3 showed a marked increase in platelet-derived cfRNA (Fig. 3B). Finally, Subgroup 4 exhibited elevated cfRNA fractions from B cells, thymocytes, kidney epithelial cells, and cardiac muscle cells (Fig. 3B). Interestingly, we found that the thymocyte fraction was strongly correlated with patient age, while B cell, kidney epithelial cell, and cardiac muscle cell fractions were not (Figure S4A-D). Furthermore, we found that the hepatocyte and kidney epithelial cell fractions were correlated in KD subgroup 1 samples but not in KD subgroup 4 samples despite both having elevated levels of kidney epithelial cell derived cfRNA (Figure S4E).

### Unsupervised clustering

We next examined whether cfRNA could identify KD subgroups *de novo*. Using the most variable and abundant transcripts, we performed unsupervised clustering and found that the samples clustered primarily on platelet fraction, likely due to platelet fraction being the most variable cell type of origin (Figure S5A). We performed principal component analysis (PCA) and found that the first principal component 1 (PC1), which accounted for 32% of the variance, correlated very strongly with platelet fraction (Figure S5B). To avoid clustering primarily on platelet fraction, we removed transcripts with high PC1 loadings. Subsequent unsupervised hierarchical clustering of this adjusted set revealed distinct clusters for subgroup 1 and subgroup 4 (Figure S5C). Subgroups 2 and 3 samples however were not separated based on the unsupervised clustering of the molecular data, possibly due to feature selection and higher variance in features unique to subgroups 1 and 4. Thus, *de novo* clustering of the molecular data identified three KD subgroups, with two groups matched with the clinically defined groups, and one group that matched a mixture of the remaining two clinically defined subgroups. Further analyses with larger sample sizes may be needed to fully explore *de novo* clustering using cfRNA.

## Discussion

**Fig. 4:**
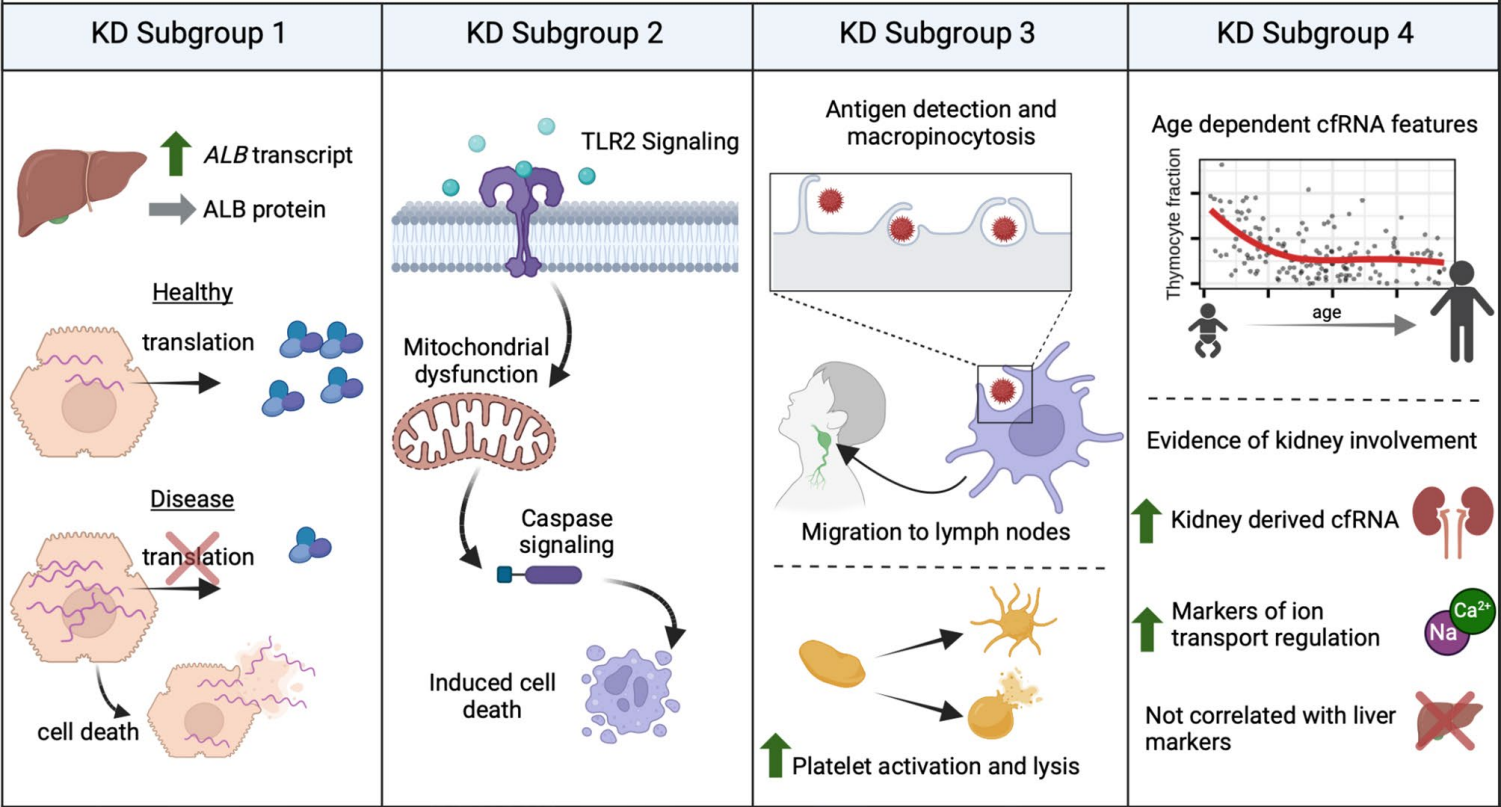
Graphical summary of findings. This figure was made using BioRender.com.

In this study, we compared previously described KD subgroups using plasma cfRNA profiling, assessing molecular differences at three levels of cfRNA information: transcript abundance, pathway enrichment, and cell type-of-origin contributions. Using supervised analyses, we detected signatures of both known and novel features that distinguish these subgroups, highlighting the breadth of information in cfRNA (Table 1; Fig. 4). Furthermore, we recapitulated two of the four subgroups using unsupervised clustering of the cfRNA samples, thus supporting a molecular basis for the presence of these subgroups. The results of this study provide novel insights into KD subgroups and highlight the utility of cfRNA for characterizing disease at the molecular level. Below, we highlight the cfRNA profiles of each subgroup, combining and interpreting the results from all three levels of analysis.

**Table 1:** Summary of key results from supervised analysis of plasma cell-free RNA from 98 acute, pre-treatment KD patients divided into subgroups based on clinical data.

Transcript	Clinical Subgroup			
	1	2	3	4
	ALB	KDSR	FLNAl	DYRK2
Pathway	**Liver-related**: - Fibrinolysis - Negative Regulation of Lipase Activity - Recycling of Bile Acids and Salts	**Antigen detection pathways**: - Toll Like Receptor 2 Signaling - Positive regulation of Interferon Alpha Production - Regulation of MHC Class II Biosynthetic Process	**Lipid biosynthesis**: **-** Cellular Lipid Biosynthetic Process - Sphingolipid De Novo Biosynthesis	**Differentiation and development**: **-** Distal Tubule Development - Collecting Duct Development - Somatic Stem Cell Division
	**Gastrointestinal tract**: - Smooth muscle cell apoptotic Process - Intestinal cholesterol absorption - Regulation of intestinal absorption	**Cell death related pathways**: - Regulation by C Flip - Caspase Activation via Death Receptors in the Presence of Ligand	**Actin-dependent functions**: - Micropinocytosis - Modification of Postsynaptic Actin Cytoskeleton - Positive Regulation of Actin Nucleation	**Ion transport related pathways**: - Positive Regulation of Calcium Ion Transport Into Cytosol - Renal Sodium Ion Absorption - Renal Sodium Ion Transport
Cell Type-of-origin	- hepatocytes - intrahepatic cholangiocytes - neutrophils	- neutrophils - myeloid progenitor cells	- platelets	- B cells - thymocytes - cardiac muscle cells

Abbreviations: ALB, albumin; KDSR1, 3-Ketodihydrosphingosine Reductase; FLNA, filamin A; DYRK2, Dual Specificity Tyrosine Phosphorylation Regulated Kinase 2.

### KD subgroup 1

Elevated liver enzymes due to liver dysfunction or damage was a hallmark feature of this subgroup, and this was mirrored across all levels of cfRNA analysis. The most abundant transcript was *ALB*, the most enriched pathway was fibrinolysis, and there was a higher contribution from liver cell types in the cfRNA transcriptomes. Although ALB protein synthesis is decreased during fever and inflammation – with serum ALB protein levels similar between KD subgroups- *ALB* cfRNA transcripts were elevated only in KD subgroup 1^3,19,20^. We hypothesize that during acute inflammation, *ALB* mRNA remains abundant in hepatocytes, but is not translated as the synthetic hepatic machinery turns to translation of acute phase reactant proteins. Thus, elevated *ALB* transcripts in plasma cfRNA directly reflect hepatocyte death. Increased neutrophil-derived cfRNA was also observed, possibly linked to liver inflammation.

### KD subgroup 2

In KD subgroup 2, we observed signatures of mitochondrial dysfunction, including initiation, metabolic dysregulation, and induction of cell death. The most enriched pathway was Toll-like receptor 2 signaling, known to initiate mitochondrial dysfunction^21,22^. This was further evidenced by elevated transcripts for *KDSR*, crucial for sphingolipid synthesis and proper mitochondrial function^23^. We also found enrichment in pathways related to glutathione and folic acid processing; dysregulation of these mitochondrial metabolites can lead to cell death^24,25^. The second most enriched pathway, Regulation of C-Flip, is associated with apoptosis due to mitochondrial dysfunction^26^. Collectively, these findings indicate that KD subgroup 2 showed signatures along the entire mitochondrial dysfunction axis. Similar patterns in febrile control groups suggest infection with a pathogen as a shared initiation factor. Lastly, elevated neutrophil-derived cfRNA may reflect increased inflammation from oxidative stress.

### KD subgroup 3

The cfRNA transcriptome of KD subgroup 3 showed signatures of increased platelet activation and dendritic cell activity. Cell type-of-origin analysis revealed higher platelet fractions compared to other subgroups, despite subgroup 4 having the highest platelet counts (Table S2)^3^suggesting greater RNA release from platelets in subgroup 3. We also identified signatures of dendritic cell activity such as macropinocytosis, including the most abundant cfRNA transcript, *FLNA*, a gene involved in ruffle formation during macropinocytosis^27^. Additionally, we observed an enrichment of genes associated with endosomal lumen acidification, a downstream effect of macropinocytosis. These findings highlight dendritic cells’ antigen-trapping processes, which may relate to the cervical lymph node enlargement observed in this subgroup.

### KD subgroup 4

The cfRNA profiles of KD subgroup 4 reflected the patients’ young age and distinct cellular and tissue involvements. We observed correlations between age and cfRNA features, such as *DYRK2* transcript abundance and thymocyte-derived cfRNA fractions, suggesting cfRNA may be a useful analyte to track early life development and further studies are needed to explore this. We also identified non-age-related features specific to KD subgroup 4 using age-matched febrile controls: high lymphocyte-derived cfRNA consistent with elevated lymphocyte counts, high cardiomyocyte-derived cfRNA aligning with the highest rate of CAA, and increased kidney epithelial cell-derived cfRNA. The relationship between this injury pattern and the shed cells found in the urine of KD patients (sterile pyuria) deserves further exploration. The renal injury findings are further supported by the enrichment of ion transport pathways in KD subgroup 4. More work is needed to explore renal involvement in KD subgroup 4 patients and the impact of age on cfRNA profiles.

We also performed unsupervised clustering on the cfRNA samples. We first removed transcripts directly related to platelet fraction to avoid clustering samples solely based on platelet contributions. We found that subgroup 1 samples clustered together, as did subgroup 4 samples, supporting the results from Wang et al.^3^. We postulate that subgroup 2 and subgroup 3 did not cluster due to inherent limitations in using cfRNA for clustering analysis. Given that cfRNA is derived from many cell types at highly variable levels, it follows that clustering is directly dependent on the differing contributions of these cell types, as we observed the dependency on platelet fraction on clustering. We observed the most distinct cell type-of-origin profiles in subgroup 1 and subgroup 4, while subgroup 2 and subgroup 3 were more similar.

Our findings suggest that KD is potentially not a single disease triggered by one agent, but comprises distinct subgroups which may have varying etiologies, aligning with previous studies^2,28^. Whether these differences are driven by environmental or genetic factors remains unclear, emphasizing the need to investigate both while considering KD subgroups. Furthermore, recognizing these subgroups may be crucial for precision medicine in patient management. For example, if subgroup 1 patients are at the highest risk for liver damage, additional therapy and monitoring might be beneficial. By considering KD subgroups in determining and testing treatment strategies, we may discover subgroup specific treatments that would not have been effective in the total KD population.

Our findings demonstrate measurable cfRNA differences among KD subtypes and motivate the development of a molecular assay to subtype KD patients. Such a test could be implemented as a sequencing-based assay or qPCR-based assay targeting a select panel of informative transcripts. Translation to clinical use will require further analyses, validation, and will need to address the limitations of the present work. Our study was conducted at a single center and multi-site validation in ethnically diverse cohorts is required to confirm the described KD subtype-specific cfRNA signatures. In addition, cfRNA profiling was performed with a research-grade next-generation sequencing workflow that requires specialized instrumentation and a multi-day day turn-around. While feasible in some hospital labs, widespread use will depend on transfer to a rapid turnaround platform (e.g. quantitative PCR).

We present molecular differences between KD subgroups and discover unique features of these subgroups previously not observed. These findings deepen our understanding of KD and suggest new paths forward to integrate subgroups into both genetic and environmental studies to understand host susceptibility and response to the triggers that initiate vasculitis.

## Methods

### Clinical cohort

We analyzed a cohort of 98 patients diagnosed with KD at Rady Children’s Hospital-San Diego from 2006 to 2021. Diagnosis was confirmed by two experienced KD clinicians (AHT and JCB) using the American Heart Association criteria for complete or incomplete KD^1^. Clinical data was prospectively recorded at the time of diagnosis (Table S1). Previous analysis of clinical and laboratory features grouped each patient into one of four subgroups (*n* = 25 KD subgroup 1, *n* = 25 KD subgroup 2, *n* = 25 KD subgroup 3, *n* = 23 KD subgroup 4) (Table S2)^3^. We further analyzed 86 samples from pediatric febrile controls (FC; *n* = 62 viral infection and *n* = 24 bacterial infection) and 5 samples from healthy children (ages 4.0-6.1 years, 1 male and 4 females). Viral and bacterial infection patients were adjudicated, and a final diagnosis was assigned 2–3 months after the acute illness to allow time for serologies, recurrence, and clinical recovery to be assessed by a research team of one ED clinician and one pediatric infectious disease expert. The FC diagnoses included viral (Adenovirus (*n* = 20), Rhino/Enterovirus (*n* = 3), EBV (*n* = 5), influenza (*n* = 3), Parainfluenza (*n* = 3), RSV (*n* = 1)), and bacterial infections (*n* = 24). Patients with a self-limited, febrile illness that resolved without treatment and for whom clinical testing was either negative or not done were classified as having “viral syndrome” (*n* = 27). Clinical data was abstracted from the medical record and entered into a REDCap database housed at UCSD^12,13^.

### Ethics

Ethical approval for this study was granted by the UCSD Institutional Review Board (UCSD # 140220), informed consent was obtained from the parents of participants, and all research was performed in accordance with relevant guidelines and regulations.

### Cell-free RNA sequencing and analysis

Pre-treatment plasma samples were collected and stored at -80 °C. cfRNA was extracted, converted into cDNA, prepared into libraries, sequenced, and analyzed as previously described^5^. We compared transcript abundance using a negative binomial model and performed variance stabilization transformation (VST) using the DESeq2 R package^14^. We conducted single sample gene set enrichment analysis (ssGSEA) using the GSVA package in R^15^ (ssgsea.norm = FALSE, tau = 0.75, min.sz = 10) using pathways from the Molecular Signatures Database (v2023.2)^16–18^. We performed cell type deconvolution as previously described^5^. For unsupervised clustering, we used gene count data after VST. First, we selected genes with the highest variance and abundance using the elbow method. We then calculated a correlation matrix, performed hierarchical clustering, and cut the dendrogram to achieve four clusters. To account for platelet fraction, we performed principal component analysis (PCA), which revealed a strong correlation between the first principal component (PC1) and platelet fraction. We then removed genes with high or low PC1 loadings using the elbow method, calculated a correlation matrix, and performed hierarchical clustering using the final gene set (*n* = 851 genes).

### Statistics

The programming language R (v4.1.0) was utilized for all statistical analyses. Statistical significance was assessed through two-sided Mann-Whitney U tests, unless specified otherwise. In boxplots, boxes denote the 25th and 75th percentiles, the band within the box signifies the median, and whiskers extend to 1.5 times the interquartile range of the hinge.

## Supplementary Information

Below is the link to the electronic supplementary material.

Supplementary Material 1

Supplementary Material 2

Supplementary Material 3

## Data Availability

De-identified RNA-seq count matrices have been uploaded to the NCBI (National Center for Biotechnology Information) GEO (Gene Expression Omnibus) database and will be publicly available upon publication (GSE255555). All code has been deposited on GitHub and will be available upon publication (https://github.com/conorloy/cfRNA_KD_subgroups).
