# Supplementary material for: Molecular characterization of Kawasaki disease subgroups using cell-free RNA profiling
Source: Sci Rep. 2025 Aug 14;15:29799. doi: 10.1038/s41598-025-15843-7 (PMC12354920; doi:10.1038/s41598-025-15843-7)
Supplement: Supplementary file 3 — Supplementary Material 3 [file 41598_2025_15843_MOESM3_ESM.docx]

**Supplementary Materials for**

**Molecular characterization of Kawasaki disease subgroups using cell-free RNA profiling**

Conor J. Loy, PhD^1^; Hao Wang, MD^2^; Jihoon Kim, PhD^3^; Chisato Shimizu, MD^2^; Joan Lenz^1^, Emma Belcher^1^, Adriana H. Tremoulet, MD^2,4^; Jane C. Burns, MD^2,4^*; Iwijn De Vlaminck, PhD^1^*

^1^ Meinig School of Biomedical Engineering, Cornell University, Ithaca, NY 14850

^2^ Department of Pediatrics, Kawasaki Disease Research Center, University of California San Diego, La Jolla, CA 92093

^3^ Department of Biomedical Informatics and Data Science, Yale School of Medicine, New Haven, CT 06510

^4^ Department of Pediatrics, Rady Children’s Hospital-San Diego, San Diego, CA 92123

*Co-corresponding authors:

Jane C. Burns (jcburns@health.ucsd.edu)

Iwijn De Vlaminck (vlaminck@cornell.edu)

|  | **KD**  (n= 98) | **FC**  (n=86) | **p-value** |
| --- | --- | --- | --- |
| **Demographic features** |  |  |  |
| Age, yrs | 3.5 (2.1-5.4) | 6.4 (4.6-8.7) | 5.28e-8 |
| Male, n (%) | 56 (57) | 51 (59) | ns |
| **Race/Ethnicity, n (%)** |  |  | ns |
| Asian | 16 (16) | 6 (7) |  |
| AA | 4 (4) | 3 (3) |  |
| White | 18 (18) | 29 (34) |  |
| Hispanic | 30 (31) | 29 (34) |  |
| > 2 races | 26 (27) | 18 (21) |  |
| Others | 4 (4) | 1 (1) |  |
| **Clinical presentation** |  |  |  |
| Illness day of plasma collection^a^ | 5 (4-7) | 6 (5-7) | ns |
| Zmax^b^ | 1.5 (1.1-2.0) | NA |  |
| Pts. with Zmax>2.5, n (%) | 11 (11) | NA |  |
| IVIG^c^ non-responder, n (%) | 16 (21) | NA |  |
| KD shock, n (%) | 3 (3) | NA |  |
| **Laboratory data:** |  |  |  |
| WBC^d^ x 10^3^/mm^3^ | 11.4 (9.2-15.4) | 9.3 (6.2-13.8) | 1.95e-3 |
| % neutrophils | 56 (45-66) | 55 (37-67) | ns |
| % bands | 11 (2-20) | 7 (3-13) | ns |
| % lymphocytes | 20 (11-29) | 27 (14-38) | 0.01 |
| ZHgb^e^ | -1.2 (-2.2 to 0.5) | -0.4 (-1.2 to 0.3) | 1.07e-4 |
| Platelet count x 10^3^/mm^3^ | 324 (241-408) | 237 (172-318) | 1.63e-6 |
| ESR^f^, mm/h | 59 (42-70) | 30 (16-44) | 7.6e-13 |
| CRP^g^, mg/dL | 6.5 (3.6-15.2) | 3.3 (1.6-5.7) | 2.4e-7 |
| ALT^h^, IU/L | 50 (26-167) | 29 (23-136) | 0.03 |
| Data are “median (Interquartile range (IQR))” or “n (N%)” unless specified. p-values were calculated using a Mann Whitney-U test or X^2^ between KD and Febrile Control. Laboratory data are pre-treatment. ns= not significant (p-value 0.05).  FC = Febrile Control. ^a^Illness Day 1= first day of fever. ^b^ Zmax: Maximum Z score (internal diameter normalized for body surface area) for the right and left anterior descending coronary arteries. ^c^ IVIG: intravenous immunoglobulin, ^d^ WBC: white blood cell count, ^e^ ZHgb: hemoglobin concentration normalized for age, ^f^ ESR: erythrocyte sedimentation rate, ^g^ CRP: C-reactive protein, ^h^ ALT: Alanine transaminase. | | | |

**Table S1. Characteristics of KD patients and febrile controls.**

| Subgroup | Main clinical feature | Additional characteristics |
| --- | --- | --- |
| 1 | Hepatobiliary involvement | Oldest, lowest risk of coronary artery aneurysms, highest risk of IVIG resistance |
| 2 | High % band neutrophils | Lowest WBC and platelet count, highest risk of KD shock |
| 3 | Cervical lymphadenopathy | Higher WBC, platelet count, erythrocyte sedimentation rate, and C-reactive protein |
| 4 | Young age at onset | Highest risk of coronary artery aneurysms, highest platelet count and percentages of lymphocytes |

**Table S2.** Characteristics of KD Subgroups based on unsupervised clustering of 14 clinical features. Adapted from Wang et al.^3^

| **Gene ID** | **Gene Symbol** | **Gene Name** | **Direction** |
| --- | --- | --- | --- |
| ENSG00000144283.22 | PKP4 | Plakophilin 4 | Up in KD |
| ENSG00000131370.16 | SH3BP5 | SH3 Domain Binding Protein 5 | Up in KD |
| ENSG00000163638.13 | ADAMTS9 | ADAM Metallopeptidase With Thrombospondin Type 1 Motif 9 | Up in KD |
| ENSG00000214357.9 | NEURL1B | Neuralized E3 Ubiquitin Protein Ligase 1B | Up in KD |
| ENSG00000146278.11 | PNRC1 | Proline Rich Nuclear Receptor Coactivator 1 | Up in KD |
| ENSG00000260314.3 | MRC1 | Mannose Receptor C-Type 1 | Up in KD |
| ENSG00000137710.17 | RDX | Radixin | Up in KD |
| ENSG00000133112.17 | TPT1 | Tumor Protein, Translationally-Controlled 1 | Up in KD |
| ENSG00000183688.4 | RFLNB | Refilin B | Up in KD |
| ENSG00000137959.17 | IFI44L | Interferon Induced Protein 44 Like | Down in KD |
| ENSG00000163131.12 | CTSS | Cathepsin S | Down in KD |
| ENSG00000168329.14 | CX3CR1 | C-X3-C Motif Chemokine Receptor 1 | Down in KD |
| ENSG00000160791.13 | CCR5 | C-C Motif Chemokine Receptor 5 | Down in KD |
| ENSG00000164054.16 | SHISA5 | Shisa Family Member 5 | Down in KD |
| ENSG00000138642.15 | HERC6 | HECT And RLD Domain Containing E3 Ubiquitin Protein Ligase Family Member 6 | Down in KD |
| ENSG00000112343.11 | TRIM38 | Tripartite Motif Containing 38 | Down in KD |
| ENSG00000127951.8 | FGL2 | Fibrinogen Like 2 | Down in KD |
| ENSG00000205413.8 | SAMD9 | Sterile Alpha Motif Domain Containing 9 | Down in KD |
| ENSG00000055130.17 | CUL1 | Cullin 1 | Down in KD |
| ENSG00000170581.14 | STAT2 | Signal Transducer And Activator Of Transcription 2 | Down in KD |
| ENSG00000089127.15 | OAS’ | 2’-5'-Oligoadenylate Synthetase 1 | Down in KD |
| ENSG00000111335.14 | OAS’ | 2’-5'-Oligoadenylate Synthetase 2 | Down in KD |
| ENSG00000133106.15 | EPSTI1 | Epithelial Stromal Interaction 1 | Down in KD |
| ENSG00000100911.16 | PSME2 | Proteasome Activator Subunit 2 | Down in KD |
| ENSG00000165949.12 | IFI27 | Interferon Alpha Inducible Protein 27 | Down in KD |
| ENSG00000185880.13 | TRIM69 | Tripartite Motif Containing 69 | Down in KD |
| ENSG00000186407.7 | CD300E | CD300e Molecule | Down in KD |
| ENSG00000130816.17 | DNMT1 | DNA Methyltransferase 1 | Down in KD |
| ENSG00000130487.9 | KLHDC7B | Kelch Domain Containing 7B | Down in KD |
| ENSG00000196664.5 | TLR7 | Toll Like Receptor 7 | Down in KD |

**Table S3. Significantly differentially abundant transcripts in all KD subgroups compared to adenovirus samples.** DESeq2, Benjamini-Hochberg adjusted p-value < 0.01, absolute fold change > 1.25.

| **Gene Symbol** | **Gene Name** |
| --- | --- |
| *APOH* | Apolipoprotein H |
| *APOC1* | Apolipoprotein C1 |
| *APOC3* | Apolipoprotein C3 |
| *TLR1* | Toll Like Receptor 1 |
| *STAT1* | Signal Transducer And Activator Of Transcription 1 |
| *JAK2* | Janus Kinase 2 |
| *TNFSF10* | TNF Superfamily Member 10 |
| *ATIC* | 5-Aminoimidazole-4-Carboxamide Ribonucleotide Formyltransferase/IMP Cyclohydrolase |
| *MTHFD2* | Methylenetetrahydrofolate Dehydrogenase (NADP+ Dependent) 2, Methenyltetrahydrofolate Cyclohydrolase |
| *GGT7* | Gamma-Glutamyltransferase 7 |
| *PEDS1* | Plasmanylethanolamine Desaturase 1 |
| *PPM1L* | Protein Phosphatase, Mg2+/Mn2+ Dependent 1L |
| *MAPKAPK2* | MAPK Activated Protein Kinase 2 |
| *WASF1* | WASP Family Member 1 |
| *NCKAP1* | NCK Associated Protein 1 |
| *KLHL3* | Kelch Like Family Member 3 |
| *DLG5* | Discs Large MAGUK Scaffold Protein 5 |
| *LBH* | LBH Regulator Of WNT Signaling Pathway |
| *CAV1* | Caveolin 1 |
| *KLHL3* | *KLHL3* |

**Table S4. Gene ID and name key.**


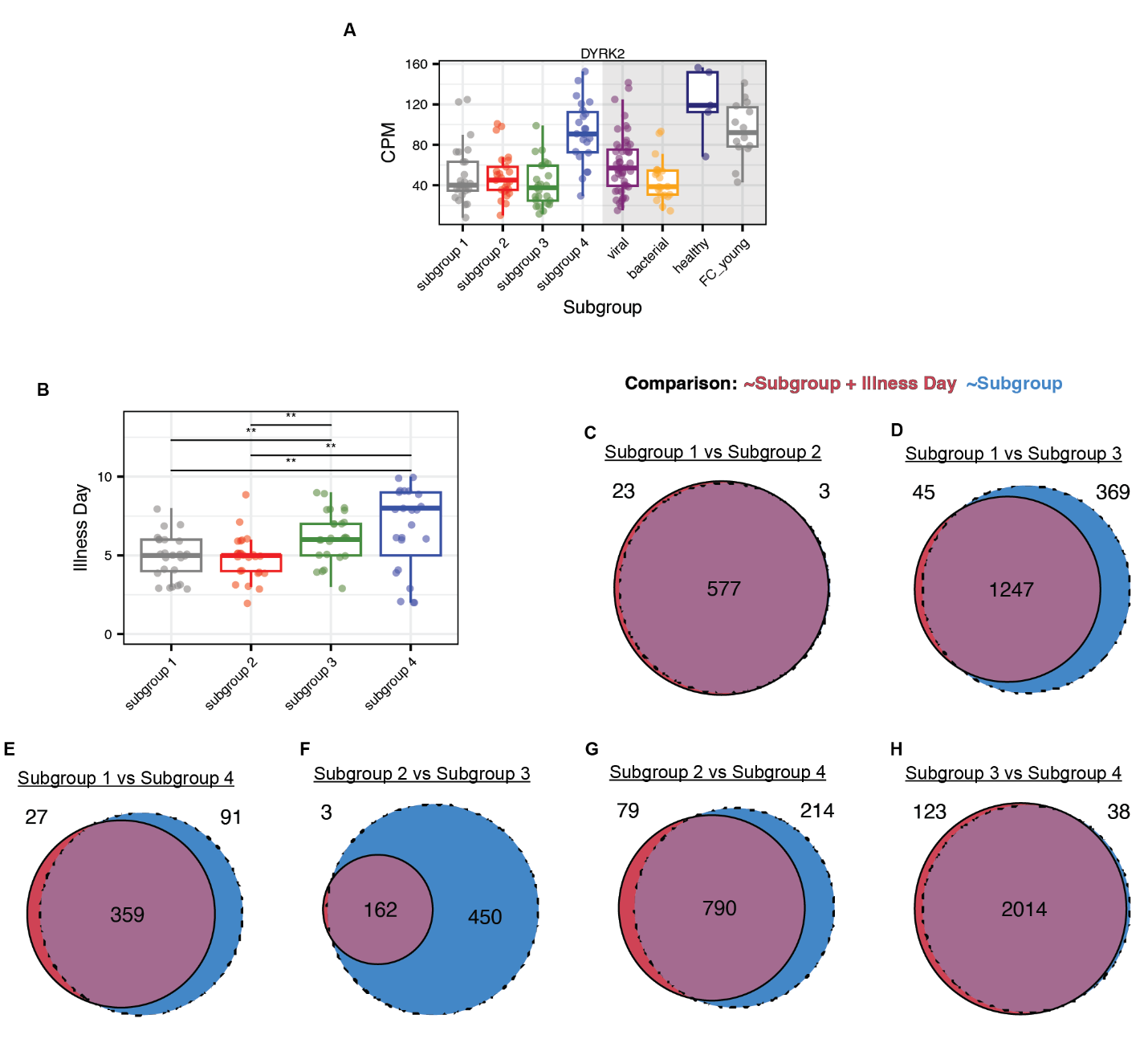


**Figure S1. Differential abundance comparisons.** (**A**) Gene transcript abundance of *DYRK2* across subgroups. “FC_young” indicates viral and bacterial patients under four years of age. **(B)** Illness day at blood draw stratified by KD subgroup. Points are vertically and horizontally jittered for visibility. Asterisks indicate statistical significance: ns, non-significant; *, p < 0.1; **, p < 0.01; ***, p < 0.001. (**C-H**) Euler diagrams summarizing the overlap of differentially abundant transcripts in each pairwise comparison modelled with and without illness day as a covariate.


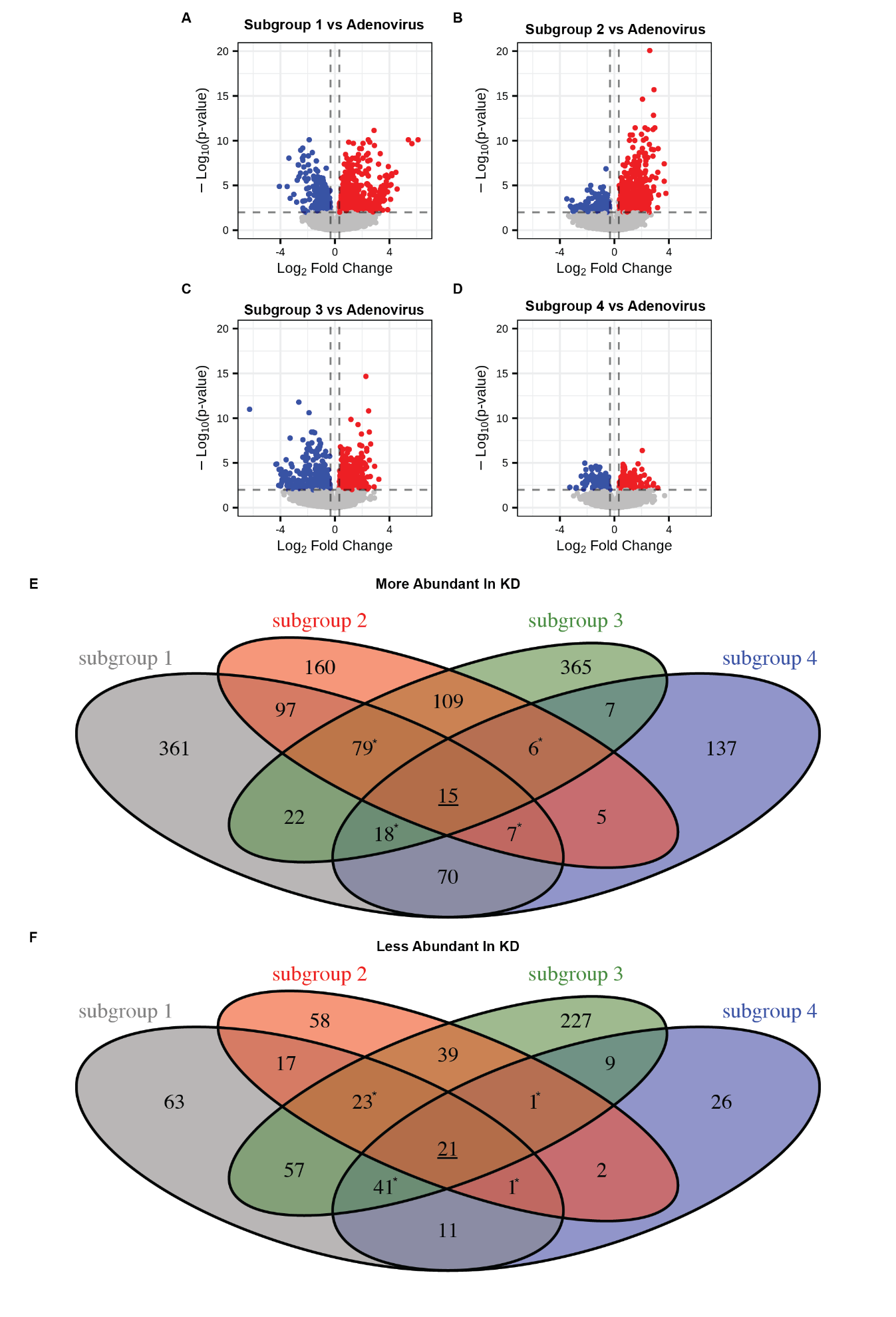


**Figure S2. Comparison to adenovirus samples.** Differential abundance analyses between KD subtypes and adenovirus samples are shown for: (**A**) Subtype 1, (**B**) Subtype 2, (**C**) Subtype 3, (**D**) Subtype 4. Volcano plots depict all RNA transcripts, with transcripts more abundant in the first listed subtype shown in red, and those less abundant in blue. Venn diagrams display the overlap of significantly differentially abundant RNA transcripts (DESeq2, Benjamini-Hochberg adjusted p-value < 0.01, absolute fold change > 1.25) between KD subgroups and patients with adenovirus infection. The number of RNA transcripts (**E**) more abundant in KD subgroups compared to adenovirus and (**F**) less abundant in KD subgroups compared to adenovirus. Underlined numbers are the count of overlapping transcripts in all four comparisons and asterisks indicate the number of overlap in three of four comparisons.


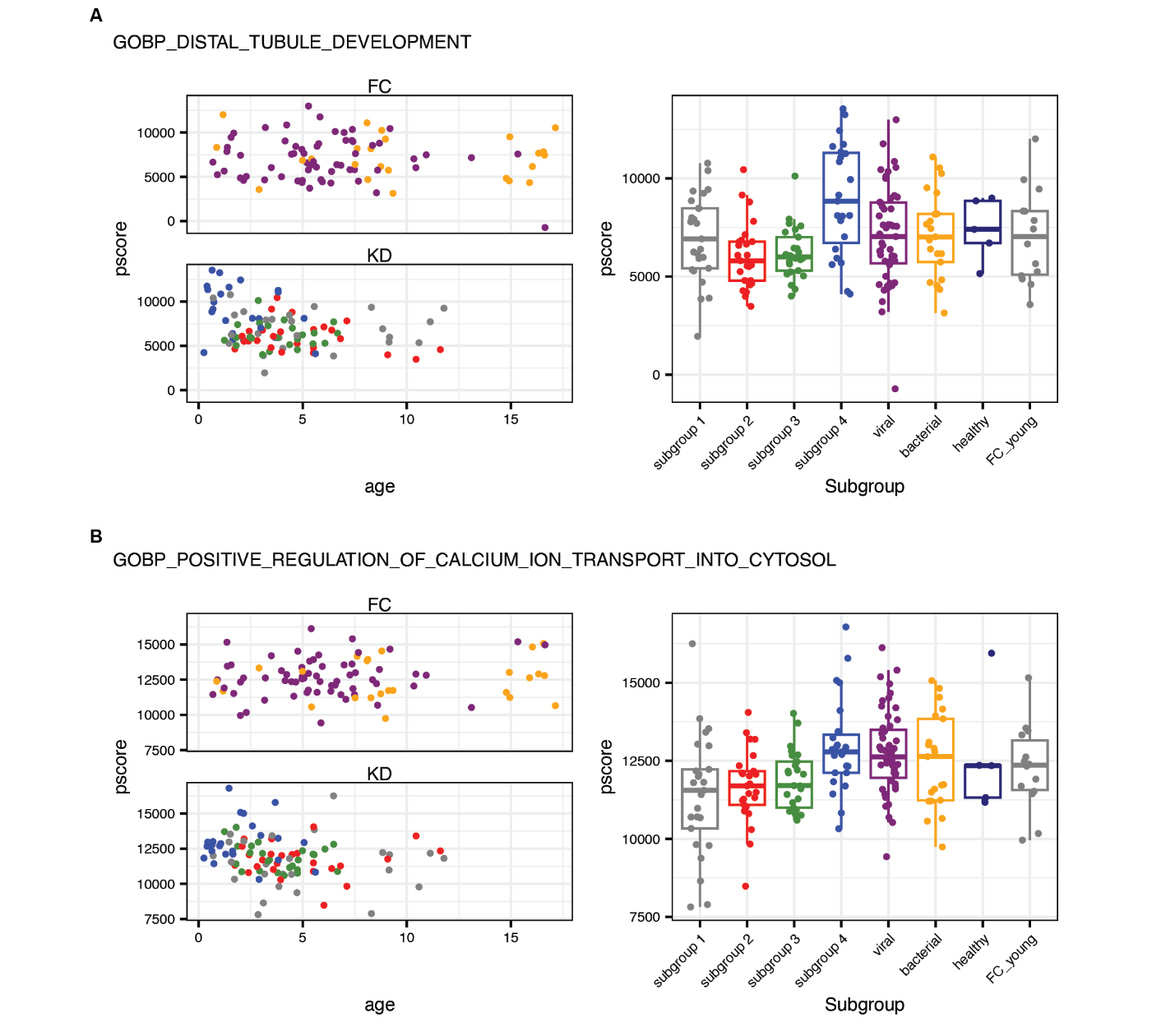


**Figure S3. Impact of age on pathway enrichment scores.** Scatterplot of age and pathway enrichment scores and boxplot of pathway enrichment scores across subgroups, with “FC_young” indicating viral and bacterial samples under four years of age for pathways (**A**) GOBP Distal Tubule Development, and (**B**) GOBP Positive Regualtion of Calcium Ion Transport into Cytosol.


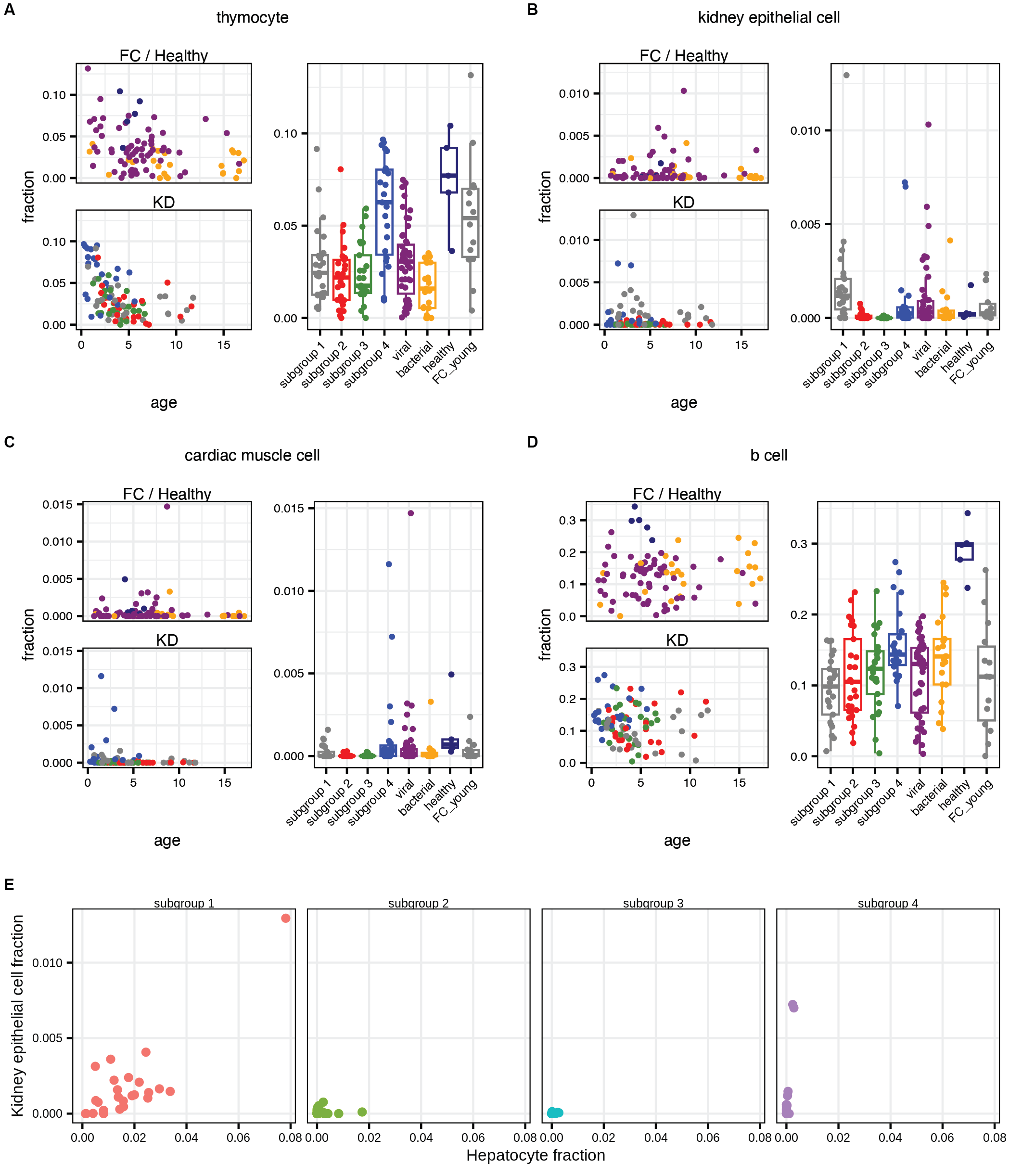


**Figure S4. Impact of age on cell type-of-origin levels.** Scatterplot of age and cell type-of-origin fractions and cell type-of-origin fractions across subgroups, with “FC_young” indicating viral and bacterial samples under four years of age for cell types: (**A**) thymocyte, (**B**) kidney epithelial cell, (**C**) cardiac muscle cell, and (**D**) B cell. (**E**) Scatter plot of hepatocyte fraction and kidney epithelial cell fraction in each KD subgroup.


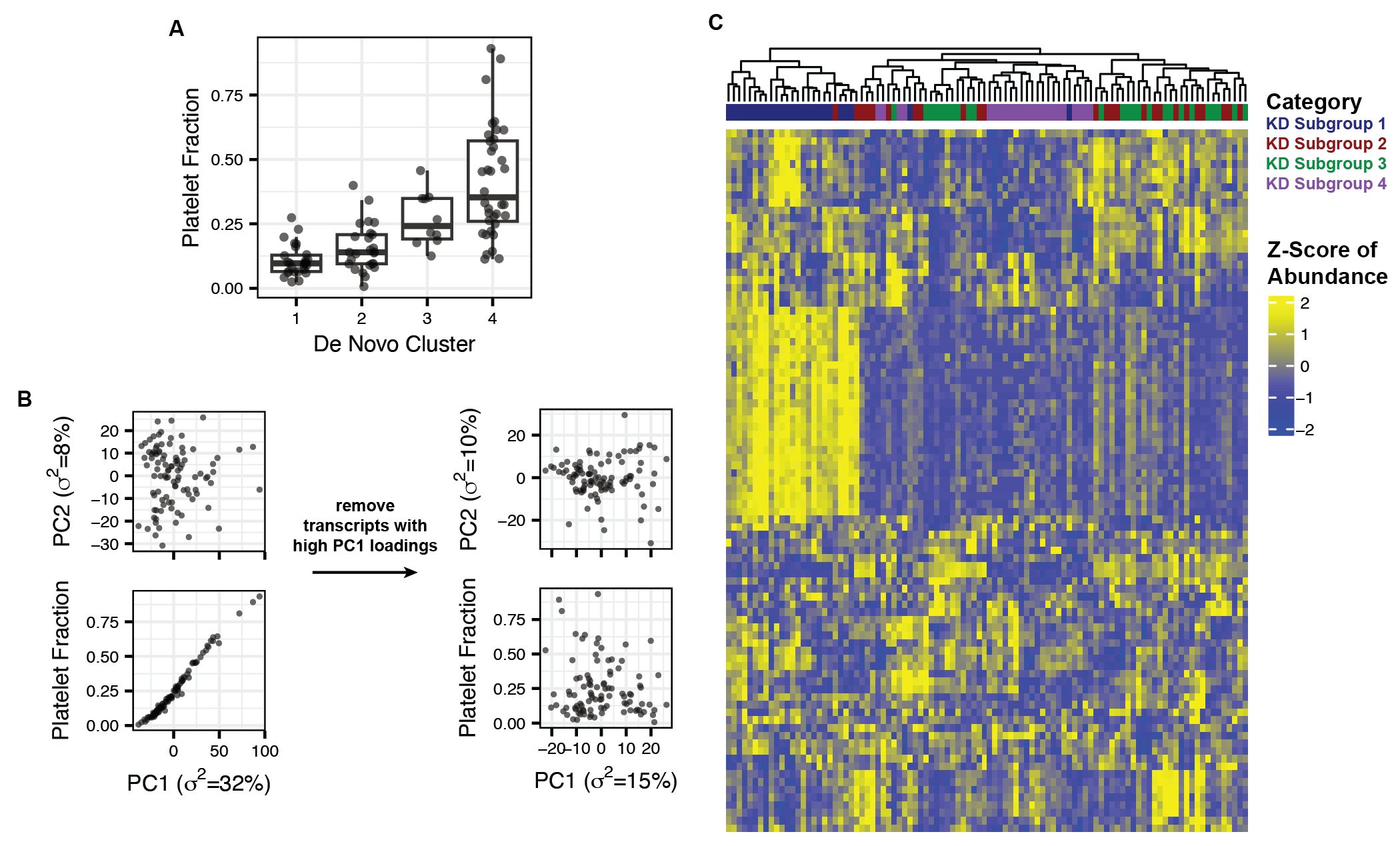


**Figure S5. Unsupervised clustering of the cfRNA data.** (**A**) Platelet fraction of sample groups hierarchically clustered using the most variable and abundant genes inclusive of platelet transcripts. (**B**) The influence of platelet fraction on clustering was mitigated by performing principal component analysis (PCA). Platelet fraction was found to be strongly correlated with the first principal component (PC1, explaining 32% of the variance), so transcripts with high PC1 loadings were removed. (**C**) Unsupervised hierarchical clustering was then applied to the most variable transcripts, with the heatmap showing Z-scores of transcript abundance across KD subgroups.

**Supplementary File 1 (separate file).** DESeq2 output tables from each KD subgroup pairwise comparison.

**Supplementary File 2 (separate file).** ssGSEA results from each KD subgroup one-versus-all comparison.
